# The Research Fellow Position: A Gateway to U.S. Residency

**DOI:** 10.1055/s-0045-1809411

**Published:** 2025-06-12

**Authors:** M-Nasan Abdul Baki, Hany Habib

**Affiliations:** 1 Department of Pathology and Laboratory Medicine, Allegheny General Hospital, Pittsburgh, Pennsylvania, United States; 2 Department of Internal Medicine, Allegheny Health Network, Pittsburgh, Pennsylvania, United States

For decades, Syrian medical graduates have sought training beyond national borders. While Germany has traditionally been preferred due to its accessible pathway and high-quality training, recent years have seen growing interest in U.S. residency programs despite significant barriers. 1 Financial constraints, the absence of Prometric testing centers in Syria, and limited access to U.S. clinical experience create significant hurdles for Syrian graduates pursuing the U.S. residency match.

Research fellowships provide a critical entry point into the U.S. medical system. Though most remain unpaid and require considerable personal sacrifice, these positions in basic, clinical, and translational medicine offer invaluable opportunities for mentorship, academic productivity, and professional networking. 2 These experiences significantly enhance applicants' competitiveness for the National Resident Matching Program, particularly for competitive specialties where American experience is highly valued. 3

This model benefits individual applicants while strengthening U.S. research through physicians who bring diverse perspectives, strong scientific inquiry, and dedication to advancing medical knowledge. Their contributions enhance research productivity within residency programs, fostering innovation and collaboration. By gaining expertise in research methodologies and clinical practice, they become positioned to contribute to Syria's health care system and research infrastructure in the future. This exchange of knowledge and experience advances global medical education and improves patient care on a broader scale.
